# Reframing sperm cryopreservation in male cancer survivors: from procedural success to meaningful utilization

**DOI:** 10.1093/oncolo/oyag111

**Published:** 2026-04-08

**Authors:** Andrea Garolla

**Affiliations:** Unit of Andrology and Reproductive Medicine & Centre for Male Gamete Cryopreservation, Department of Medicine, University Hospital of Padova, Padova, 35128, Italy

## Abstract

The expert review by Alexander and colleagues addresses a long-standing yet insufficiently interrogated paradox in contemporary oncofertility item: While sperm cryopreservation prior to gonadotoxic cancer therapy is widely endorsed, technically straightforward, and increasingly accessible, its subsequent utilization by male cancer survivors remains consistently low. This discrepancy, persisting across institutions, health-care systems, and decades, suggests that the challenge is no longer primarily technical or educational, but structural, temporal, and psychosocial in nature. The central merit of this review lies in its deliberate shift away from a narrow, procedure-centered conception of fertility preservation toward a longitudinal, survivorship-oriented framework. In doing so, the authors implicitly challenge the field to reconsider what constitutes “success” in oncofertility. If success is defined merely as successful banking prior to treatment, then current practice may appear satisfactory. If, however, success is defined as enabling informed, autonomous, and timely reproductive decision-making across the survivorship trajectory, then the low utilization rates described in this review represent a clear failure of care continuity.

## Beyond the moment of diagnosis: fertility preservation as a longitudinal process

Historically, fertility preservation in male oncology patients has been anchored to the time of diagnosis, a moment characterized by urgency, emotional overload, and compressed decision-making. This temporal concentration ensured that clinical management pathways, counseling models, and methods for evaluating the result were created. Alexander et al.^1^ convincingly argue that such an approach is intrinsically limited. Sperm cryopreservation marks the beginning but not the culmination of a reproductive care pathway whose relevance may only fully emerge years later. The review’s synthesis of utilization data is particularly instructive. Across cohorts, fewer than 10% of men who bank sperm ultimately use it for conception, despite long-term storage rates that approximate 70%-80% of cases. Importantly, this divergence cannot be simplistically attributed to recovery of spermatogenesis. Many survivors who regain adequate semen parameters still retain their cryopreserved samples for extended periods, suggesting that utilization decisions are influenced by other factors, well beyond biological necessity. This observation has important conceptual implications in the actual scenario. Cryopreserved sperm functions not only as a biological resource but also as a psychological and symbolic tool, representing future possibility, safety in time of uncertainty, and a way to preserve one’s identity over time. The value of sperm banking, therefore, cannot be fully captured by considering only parameters of its use. At the same time, the persistence of a large number of unused samples highlights the missed opportunities and the need to provide a structured follow-up together with re-evaluation and counselling programs.

## The Oncofertility Patient Care Pathway: from abstraction to operational tool

One of the most compelling contributions of the review is the proposed Oncofertility Patient Care Pathway. Rather than presenting fertility preservation as a single intervention, the pathway identifies different timepoints at which patients are particularly vulnerable to disengagement, misperception, or decisional paralysis. These include the post-treatment fertility return visit, periods of delayed readiness for family building, and moments requiring explicit decisions regarding continued storage or disposition. The fertility return visit, recommended at 12-24 months after treatment completion, deserves special attention. Although endorsed by professional societies, it remains inconsistently implemented in routine practice. The authors highlight that up to one-third of patients do not attend post-treatment fertility consultations, a finding that should prompt critical reflection on current survivorship models. Expecting patients, most often very young, at a psychologically difficult time and dealing with complex health challenges, to independently initiate fertility follow-up is unrealistic and highly unlikely. From a clinical perspective, the fertility screening visit should be reconsidered as a fundamental step in the reassessment of the patient’s general health, similar to oncology surveillance visits. Its purpose extends beyond semen analysis. It should provide a structured opportunity to reassess reproductive potential, assess testicular reserve, evaluate hormonal status, and rule out hypogonadism and associated comorbidities. Furthermore, it should provide a time to clarify misconceptions about assisted reproduction, discuss genetic and partner-related considerations, and recalibrate reproductive goals in light of changing life circumstances.

## Psychosocial determinants of non-utilization

One of the review’s key points of interest is its detailed analysis of psychosocial barriers, which are too often overlooked or relegated to secondary status in the discourse on fertility preservation. Cancer survivorship is frequently accompanied by anxiety, fear of recurrence, altered body image, and shifts in self-perception, all of which may profoundly influence reproductive decision-making. Concerns regarding masculinity, sexual function, and genetic transmission of disease may further delay or even hinder the launch of assisted reproductive technologies. Importantly, these factors are not static. Readiness for family building often evolves over time and may lag significantly behind physical recovery. The authors appropriately caution against interpreting delayed utilization as disinterest or indecision. Instead, delayed readiness should be recognized as a normative survivorship pattern, one that requires longitudinal support rather than episodic counseling. In European health-care contexts, including Italy, these psychosocial dimensions may be further shaped by cultural attitudes toward medically assisted reproduction and by legal frameworks that impose additional constraints. While the review is grounded primarily in the US system, its conceptual insights are broadly transferable and underscore the need for context-sensitive implementation.

## Financial and structural barriers: the hidden costs of survivorship

Financial toxicity emerges in the review as a persistent and under-addressed determinant of utilization. While the initial cost of sperm banking is relatively modest, cumulative storage fees and the high costs associated with assisted reproduction can represent significant barriers, particularly for young survivors early in their professional lives. Although insurance coverage for these procedures is available in some countries, it is often inconsistent, exacerbating this challenge, and introducing inequalities that disproportionately affect socioeconomically disadvantaged patients. The authors’ call for policy-level interventions is therefore well founded. However, financial considerations also intersect with clinical communication. Patients may overestimate the costs or underestimate the success rates of assisted reproductive techniques assisted reproductive techniques (ART), leading to premature abandonment of reproductive options. Transparent, repeated, and tailored financial counseling should be considered an integral component of oncofertility care rather than an ancillary service.

## Posthumous reproduction: ethical complexity and clinical responsibility

The review’s treatment of posthumous sperm use is both timely and measured. The finding that a substantial proportion of patients express preferences regarding posthumous reproduction highlights the ethical responsibility of clinicians to address this issue proactively. Deferring such discussions may leave surviving partners and families facing uncertainty, distress, and legal obstacles at moments of profound vulnerability. While legal frameworks governing posthumous reproduction vary widely across jurisdictions, the ethical principle remains consistent: Patient autonomy is best respected through anticipatory, explicit consent processes. In my opinion, the authors appropriately advocate for clear institutional policies and documentation, recognizing that posthumous considerations are not peripheral but integral to comprehensive fertility preservation counseling.

## Implications for clinical practice and research

Alexander et al. ultimately argue for a reconceptualization of oncofertility as a sustained clinical relationship rather than a time-limited intervention. This perspective aligns with broader trends in survivorship care, emphasizing continuity, multidisciplinary collaboration, and patient-centered outcomes. Future research should move beyond descriptive utilization rates to evaluate the impact of structured pathways on decisional satisfaction, psychological well-being, and long-term reproductive autonomy. Prospective implementation studies, particularly in publicly funded health-care systems, would be especially informative. Additionally, greater attention should be paid to patient-reported outcomes that capture the subjective value of fertility preservation independent of utilization.

## Conclusion

In conclusion, this expert review makes a substantial contribution to the field of oncofertility by illuminating the gap between technical success of male fertility preservation, posthumous use of gametes, and meaningful reproductive outcomes. By articulating the structural, psychosocial, and temporal barriers that impede utilization of cryopreserved sperm, and by proposing a pragmatic care pathway, the authors provide both conceptual clarity and actionable guidance. The message for clinicians and health-care systems is unequivocal: Fertility preservation does not end with cryopreservation. Only through sustained follow-up, proactive counseling, and integration of reproductive care into survivorship pathways, the full promise of oncofertility can be realized. In this sense, the review by Alexander et al. represents not only an expert synthesis, but also a call to redefine standards of care for male cancer survivors.
